# Unprecedented Polymicrobial Bacteremia With Nine Microorganisms in a Critically Ill 95% Burn Patient

**DOI:** 10.7759/cureus.93651

**Published:** 2025-10-01

**Authors:** Alexander Johnson, Julio Nasim, Brian B Draper, Cindy L Austin

**Affiliations:** 1 College of Osteopathic Medicine, Kansas City University, Kansas City, USA; 2 Emergency Medicine, NewYork-Presbyterian Brooklyn Methodist Hospital, Brooklyn, USA; 3 Infectious Disease, Mercy Hospital, Springfield, USA; 4 General and Trauma Surgery, Mercy Hospital, Springfield, USA; 5 Trauma and Burn Research, Mercy Hospital, Springfield, USA

**Keywords:** burn injury, infection control measures, polymicrobial bacteremia, rare case, severe sepsis

## Abstract

This report describes the case of a 21-year-old male with 95% total body surface area full-thickness burns who developed polymicrobial bacteremia involving nine distinct bacterial species, the highest number of diverse bacterial species documented to simultaneously coexist in a patient’s bloodstream. Despite aggressive surgical, antimicrobial, and multidisciplinary interventions, the patient succumbed to sepsis after 12 days. The case highlights the diagnostic and therapeutic challenges of managing complex infections in burn patients and underscores the critical importance of early detection, prompt treatment, and coordinated care. A literature review of the identified pathogens provides context and reveals notable findings relevant to this unprecedented case.

## Introduction

A large body of evidence demonstrates that infection is the most common contributor to morbidity and mortality in burn patients [1]. Studies looking at mortality have indicated that between 42%-65% of deaths in burn victims are attributable to infection [2]. The initial problem arises from the complete breakdown of the skin’s natural barrier, paired with an insult from a systemic inflammatory response, often in the setting of invasive procedures that together leave patients increasingly vulnerable to bacterial inoculation.

Polymicrobial infections are caused by combinations of bacteria, fungi, viruses, and parasites, with increasing awareness of frequency. In these infections, the presence of one microorganism generates an opportunistic environment for other pathogenic microorganisms to colonize [3]. Due to the exposure of the vulnerable wound environment, burn patients have higher rates of polymicrobial bacteremia than other patient populations.

Polymicrobial bacteremia is well documented in the literature among burn patients; however, most cases are reported with only two organisms, and limited cases have been reported with three to four organisms [4,5]. Remarkably, this case is a burn patient’s blood culture with growth of nine different organisms. These micro-organisms include *Staphylococcus aureus*, *Enterococcus casseliflavus*, *Stenotrophomonas maltophilia*, *Staphylococcus epidermidis*, *Enterococcus faecium*, *Acinetobacter baumannii*, *Enterobacter cloacae*, *Escherichia coli*, and *Candida albicans*. To our knowledge, based on an extensive literature review, this case represents the highest number of organisms reported simultaneously in a single patient.

## Case presentation

A 21-year-old immunocompetent, Caucasian male with no significant past medical history was admitted to a tertiary care center after sustaining extensive full-thickness burns in a house fire. Details regarding the circumstances of the incident were unclear. The patient was transported by ambulance due to weather conditions not permitting flight. He presented in critical condition with over 95% total burn surface area (TBSA), inhalation injury with carbonaceous material in the tertiary bronchi, and cardiac arrest.

Upon admission, the patient was intubated due to respiratory distress, and bronchoscopy revealed heavy secretions in the airways. Throughout the hospitalization, the patient underwent multiple surgical interventions, including open laparotomy as well as critical care interventions, including, but not limited to, central catheter placement, urinary catheter placement, parenteral nutrition, and mechanical ventilation, which are all independently associated with increased potential for nosocomial infection. Empiric antibiotic therapy was initiated due to concerns for superimposed bacterial infection in the setting of fever.

Two sets of blood cultures were obtained on admission and were negative. On hospitalization day eight, the patient became neutropenic with clinical evidence of septic shock. At that time, repeat cultures were obtained. Given the clinical picture consistent with septic shock, lack of skin integrity, and concordance between cultures, the findings were more consistent with infection and not extreme contamination of the cultures. On day nine, two additional sets of blood cultures were obtained from the right groin 30 minutes apart. This was done as the patient had minimal remaining intact skin. Sputum cultures with Gram stain were obtained from suctioning of the patient’s endotracheal tube, and bacterial and fungal wound cultures with Gram stain were taken from the back. In addition to traditional cultures, one blood culture and the sputum were tested by polymerase chain reaction (PCR). Given concordance across the blood cultures with the employment of both traditional and PCR techniques, none of these is suspected to represent contamination.

The blood culture panel revealed a complex polymicrobial infection with the presence of *E. faecium*, *S. aureus*, *S. epidermidis*, *A. calcoaceticus*/*baumannii* complex, *S. maltophilia*, *E. casseliflavus*, *E. cloacae* complex, *E. coli*, and *C. albicans*. The blood culture pathogen PCR test was repeated twice. Additionally, the presence of the *mecA/C* gene suggested presumptive methicillin (oxacillin) resistance; further subculture confirmed the diagnosis in multiple cultures, which was confirmed by the microbiology laboratory. Blood culture pathogen revealed complex polymicrobial infection (Table 1).

**Table 1:** Blood culture pathogen PCR panel. PCR = polymerase chain reaction

Pathogen	Result
*Enterococcus faecium* by PCR	Detected
*Staphylococcus aureus* by PCR	Detected
*Stenotrophomonas maltophilia* by PCR	Detected
*Enterobacter cloacae* complex by PCR	Detected
*Escherichia coli* by PCR	Detected
*Candida albicans* by PCR	Detected
*Staphylococcus epidermidis* by PCR	Detected
*Acinetobacter calcoaceticus*/*baumannii* complex by PCR	Detected
*Enterococcus casseliflavus* by blood culture	Positive

In addition to the established common medications, i.e., intravenous (IV) meropenem (administered at 1,000 mg every eight hours), IV daptomycin (prescribed at 8 mg/kg every 24 hours), IV trimethoprim (TMP)/sulfamethoxazole (given at 300 mg of TMP every eight hours, totaling 12 mg/kg TMP component daily), IV micafungin (administered at 100 mg daily), IV ampicillin sulbactam was incorporated, with a dosage of 18 g daily. The patient’s antibiotic treatment plan was meticulously crafted through a collaborative effort between infectious disease specialists and pharmacy professionals, which encompassed a multifaceted regimen to combat the complexity of their condition.

When septic shock was suspected, two sets of blood cultures were obtained, which were positive for *E. coli*, *E. faecium*, *A baumanni*, *E. cloacae*, *E. classeliflavus*, *S. epidermidis*, and *S. aureus*. PCR testing of the blood noted the same previously listed organisms with additional detection of *S. maltophilia* and *C. albicans*. A sputum culture grew *E. cloacae*, *S. maltophilia*, and *S. aureus*, while PCR of the same sample detected *E. cloacae*, *S. aureus*, and *A. baumanni*. Lastly, wound culture from the back grew three separate types of Gram-negative rods, Gram-positive cocci, and *C. albicans*.

Even with the concerted efforts of the healthcare team, the patient’s prognosis remained guarded. The development of sepsis with pan-positive blood cultures indicated a severe systemic infection, highlighting the challenges associated with managing burn-related complications in critically ill patients. Despite aggressive management efforts, including antimicrobial therapy and supportive care, the patient’s condition deteriorated, ultimately resulting in their passing after 12 hospital days. Informed consent and permission were obtained from the patient’s family for the publication of this case report.

## Discussion

An in-depth literature review of the studies involving the microorganisms in burn patients revealed notable findings relevant to this unprecedented case.

*Enterobacter cloacae* bacteremia

Only three other burn studies have been reported in the literature involving *E. cloacea* bacteremia from 1978 and 1982 [6,7] and 2017 [8]. An outbreak of bacteremia due to *E. cloacae* from 1977 to 1979 occurred in a hospital that identified *E. cloacae* as a prominent pathogen in their burn units. Most patients had multiple sites (urine, wound, sputum) colonized before bacteremia presented, and the origin of contamination from personnel or environmental items was not identified. *E. cloacae* tends to contaminate medicinal and intravenous hospital materials; therefore, vigilance of potentially infected devices, particularly in the bloodstream, is warranted [6]. A review of 15 patient cases found that the development of bacteremia was significantly related to burn center admission and severity of third-degree burns, with spread caused by the hands of staff and cross-contamination of hydrotherapy [7]. More recently, Kanamori et al. (2017) described a three-year outbreak due to *Klebsiella pneumoniae* carbapenemase-producing *E. cloacae* and *Klebsiella pneumoniae* at a burn center. This study outlines the difficulties in infection prevention and control at the burn center, emphasizing the rigorous implementation of strict standards and contact precautions for patients [8]. No publications were found involving *E. casseliflavus* bacteremia specifically in burn patients.

Acinetobacter baumannii

A two-year cohort study found that burn patients, especially those receiving hydrotherapy, are at an increased risk of *A. baumannii* bacteremia. Immunosuppression was associated with a threefold increase in risk, explaining the higher infection rates in burn patients. Additionally, respiratory failure and invasive procedures also conferred a threefold increased risk of *A. baumannii* bacteremia, which is common in burn patients [9]. An observational study evaluating 594 patients with *A. baumannii *bacteremia found that 21% of the cases of bacteremia were polymicrobial, with the most common co-pathogens being *K. pneumoniae*, *P. aeruginosa*, *E. faecium*, and *S. aureus*, of which the last two were co-pathogens in our patient. Burn injury was the only identified independent risk factor for polymicrobial bacteremia, with 43% of burn patients developing polymicrobial bacteremia. Common sources of *A. baumannii* bacteremia include respiratory infection and central line infection, with soft tissue infection also generally implicated in cases of polymicrobial bacteremia [10].

Candidemia

A literature review encompassing over 8,000 reported cases of candidemia revealed a high incidence of synchronous bacteremia in patients with candidemia, similar to what was seen in our patient. However, the patient demographic in the study did not specifically include burn injury [11].

Polymicrobial bacteremia

As referenced earlier, a large body of literature is established on polymicrobial bacteremia among burn patients, yet the vast majority of cases involve only two organisms, with relatively rare reports of cases involving three or four organisms [4,5,12]. A recent study of 114 patients with polymicrobial bacteremia showed 93.8% had *K. pneumoniae* plus one other organism, 5.3% had *K. pneumoniae* plus two other organisms, and only one patient, representing 0.9%, had *K. pneumoniae* plus three other organisms [5]. While our patient did not have *K. pneumoniae*, 20% of the patients included in the analysis were in the burn unit. This helps illustrate the severity and rarity of our patient having co-infection with nine distinct organisms.

One multicenter study with similar patient demographics as presented in our case evaluated bloodstream infections in 177 patients, with a median TBSA of 95% and inhalation injury present in 97.2% of patients. Of the 120 patients who developed bloodstream infection, 66 were polymicrobial. *A. baumannii*, *K. pneumoniae*, and *Candida* were the most common pathogens identified in the study. Likewise, our patient had both *A. baumannii* and *Candida* with 95% TBSA and inhalational injury. This observational study demonstrated the prominent occurrence of bacteremia, polymicrobial bacteremia, and recurrent bacteremia in burns with inhalation injury [13]. Although polymicrobial bacteremia is prevalent in burns, in contrast, one study that looked at 212 burn patients with suspected sepsis did not find any cases of polymicrobial bacteremia [14]. Relevant studies on this unique case are summarized in Table 2, which presents key findings on microorganisms identified in burn patients (Table 2).

**Table 2:** Relevant bacteremia burn studies. TBSA = total burn surface area

Author	Year	Organisms	Notes
Zheng et al. [4]	2020	Out of 348 (15.5%) of S. aureus bacteremia cases, 54 were polymicrobial. Of the 54 cases, 7 (13%) involved 3 organisms and 47 (87%) involved 2 organisms	Burn injury was an independent risk factor for polymicrobial bacteremia, but this study was not exclusive to burn patients
Song et al. [5]	2021	Out of 114 patients with polymicrobial bacteremia, 1 (0.9%) had 4 organisms, 6 (5.3%) had 3 organisms, and 107 (93.9%) had 2 organisms	This study enrolled 818 patients with K. pneumoniae bacteremia, of whom 114 had polymicrobial bacteremia. 52 patients were admitted for burn injury, with 23 (44%) having polymicrobial bacteremia. The study did not specify if the cases involving 3-4 organisms occurred in burn patients
Qian et al. [10]	2022	Out of 68 burn patients with bacteremia, 29 (43%) were polymicrobial (defined as 2 or more organisms). Out of the 126 total patients with polymicrobial bacteremia, 106 (84.1%) had 2 organisms and 20 (15.9%) had 3 organisms	This study enrolled patients with A. baumannii bacteremia and was not exclusive to burn patients
Zorgani et al. [12]	2010	Out of 430 cases of bacteremia in burn patients, 52 (12.1%) were polymicrobial. Of the 52 polymicrobial infections, 44 (84.6%) involved 2 organisms and 8 (15.4%) involved 3 organisms	-
Tang et al. [13]	2018	66 out of 120 (55%) cases of bacteremia in burn patients were polymicrobial, but the specific number of organisms was not specified	177 burn victims were enrolled after an industrial accident in China. Median TBSA was 95% and inhalation injury was present in 97.2% of patients
Ronat et al. [14]	2014	No cases of polymicrobial bacteremia in 212 burn patients with suspected sepsis	-

Antibiotic prophylaxis in burn patients

Invasive infection in burn patients is one of the most common complications leading to death and morbidity. Despite infection being the primary cause of death in burn patients, the prophylactic use of systemic antibiotics is not recommended, even for severe burns. The stance of the Infectious Diseases Society of America on this issue remains undetermined. While existing literature presents conflicting findings, the prevailing consensus advises against this practice at present [15,16]. Further research is necessary to offer clarity and guidance in this area. Managing extensive burn injuries poses numerous challenges, including the heightened risk of infection and sepsis. In this case, the patient’s clinical course was complicated by the onset of sepsis, as indicated by pan-positive blood cultures suggestive of a polymicrobial infection.

The Cochrane Review provides the most in-depth review up until 2013 on the safety and efficacy, and found essentially no role for antibiotic prophylaxis in burn wound infections. The authors identified 36 randomized controlled trials that examined topical antibiotics, routine systemic antibiotics, preoperative systemic antibiotics, systemic antibiotic prophylaxis upon hospital admission, nonabsorbable antibiotics, and antibiotics administered via the airway [15]. A more recent systematic review of 19 publications criticized the Cochrane Review for being too strict with inclusion and exclusion criteria and reported a limited role for antibiotic prophylaxis in certain circumstances, namely, patients who are mechanically ventilated and those receiving split-thickness skin grafts [17].

Based on the reported possible benefit of antibiotics in patients who are mechanically ventilated, as in this patient with inhalational injury and mechanical ventilation, there appears to be some weak evidence for antibiotic prophylaxis in patients requiring mechanical ventilation and those who have inhalational injury. However, this evidence is based on lower-quality studies with relatively small sample sizes that would not warrant changing practice. There were a few studies in pediatric populations that were not addressed, given that this article describes an adult case.

A retrospective single-center study involving 157 patients with 30%-60% TBSA burns suggested that routine antibiotic prophylaxis is unnecessary but did acknowledge potential benefits for patients with inhalation injury and pneumonia [16]. On our appraisal of the literature, the study design and inclusion criteria raise questions, and the article has potential serious limitations. In a nonrandomized retrospective observational study using a national database, severe burn patients on mechanical ventilation who received prophylactic systemic antibiotics had significantly lower 28-day in-hospital mortality compared to the control group [18].

Treatment considerations

Selective digestive decontamination (SDD) is a classic four-component protocol using decontamination of the digestive tract, which has been proven to prevent severe infections and reduce mortality in critically ill patients [19]. A double-blind, randomized controlled trial examining 107 burn patients with >20% TBSA and suspected inhalation injury found SDD was associated with statistically significant lower intensive care unit and in-hospital mortality and risk of pneumonia [20]. Our patient did not meet this inclusion criterion; therefore, he did not receive SDD, but this intervention may be of consideration in similar patients.

The management of sepsis in burn patients requires a multifaceted approach, including prompt administration of broad-spectrum antibiotics, supportive care, and close monitoring of clinical parameters. In this case, an infectious disease consultation was sought for further evaluation and management of the septic episode. Adjustments to the antibiotic regimen were made based on culture sensitivities, with a focus on targeting the identified pathogens.

## Conclusions

This unique case underscores the complexities inherent in managing severe burn injuries, particularly when complicated by sepsis and multiorgan dysfunction. It highlights the importance of early detection, proactive treatment, and collaborative teamwork in improving outcomes for patients struggling with severe burn injuries. Moreover, it emphasizes the skin’s crucial role as a protective barrier against multiple bacterial and fungal pathogens, noting the rarity of encountering nine different organisms simultaneously in humans.
